# Haploidentical Transplantation in Children with Acute Leukemia: The Unresolved Issues

**DOI:** 10.1155/2016/3467672

**Published:** 2016-03-24

**Authors:** Sarita Rani Jaiswal, Suparno Chakrabarti

**Affiliations:** ^1^Department of Blood and Marrow Transplantation, Dharamshila Hospital and Research Centre, Vasundhara Enclave, New Delhi 110096, India; ^2^Manashi Chakrabarti Foundation, Kolkata, India

## Abstract

Allogeneic hematopoietic stem cell transplantation (HSCT) remains a curative option for children with high risk and advanced acute leukemia. Yet availability of matched family donor limits its use and although matched unrelated donor or mismatched umbilical cord blood (UCB) are viable options, they fail to meet the global need. Haploidentical family donor is almost universally available and is emerging as the alternate donor of choice in adult patients. However, the same is not true in the case of children. The studies of haploidentical HSCT in children are largely limited to T cell depleted grafts with not so encouraging results in advanced leukemia. At the same time, emerging data from UCBT are challenging the existing paradigm of less stringent HLA match requirements as perceived in the past. The use of posttransplantation cyclophosphamide (PTCY) has yielded encouraging results in adults, but data in children is sorely lacking. Our experience of using PTCY based haploidentical HSCT in children shows inadequacy of this approach in younger children compared to excellent outcome in older children. In this context, we discuss the current status of haploidentical HSCT in children with acute leukemia in a global perspective and dwell on its future prospects.

## 1. Introduction

Despite marked improvement in the outcome of children with acute leukemia with first-line chemotherapy, a significant proportion of patients require allogeneic hematopoietic stem cell transplantation (HSCT) either in first remission (CR1) or beyond. In the BFM 95, about 12% of children diagnosed with acute lymphoblastic leukemia (ALL) went on to receive an allogeneic HSCT and the number increased in subsequent studies with introduction of MRD based risk stratification [1]. Likewise in the trials involving children with acute myeloid leukemia (AML), up to 30% of patients underwent an allogeneic transplantation [2]. In addition, allogeneic HSCT is the preferred modality of intervention beyond CR1. Thus, a conservative estimation would be that 25% of children with ALL and 40% of those with AML might require an allogeneic HSCT either in CR1 or beyond.

HLA matched family donor (MFD) remains the donor of choice in any indication for allogeneic HSCT. But with restricted family sizes, the chances of obtaining a MFD for a child are substantially reduced. Thus, alternate donor HSCT would be needed for the majority when an allogeneic HSCT is indicated and the focus of the transplant community in the past two decades has been on development of alternate donor sources.

## 2. The Dilemma of HLA Matching: Time for Cord Blood As Well

Developments in unrelated donor registries for both marrow and cord blood repositories have enabled progress in the field of allogeneic HSCT. Initial registry based studies had established equivalence between a mismatched unrelated cord blood transplantation (UCBT) and matched unrelated donor (MUD) transplantation [3]. HLA matching based on high resolution typing has improved the outcome of MUD transplants over the last two decades [4]. The limitations of North American and European registries in providing 8/8 HLA matched donors beyond the White Europeans have been largely addressed by the availability of ≥4/6 HLA matched UCB units from the existing public cord blood banks [5]. Whilst low resolution typing for HLA-A and HLA-B and high resolution typing for DRB1 were deemed optimal for UCBT aiming for 4–6/6 HLA matched units, recent studies have challenged this notion [6–9]. A retrospective analysis on 803 patients, mostly children, showed the importance of HLA-C matching to reduce transplant related mortality (TRM), which was hitherto considered redundant [6]. At the same time, high resolution allele level matching for both single and double cord units was shown to reduce TRM [7, 9]. The impact of allele level or extended HLA-C matching was shown to be independent of the cell dose. These findings, if taken to cognizance, would restrict the availability of suitably matched UCB such as ≤2 allele level mismatches including HLA-C. Thus, the attempts at optimizing the outcome of UCBT have pushed the quest for the third alternative that is HLA-haploidentical family donor (HFD) to the fore [7, 8].

## 3. Haploidentical Family Donor: Always Present but Barely Noticed until Now

The success of HSCT depends on establishment of bidirectional tolerance and compatibility of major HLA antigens is a prerequisite for the same. It has been aptly documented in the setting of unrelated donor HSCT that with each additional mismatch in HLA-A, HLA-B, HLA-C, or DRB1 the survival decreases by 10–20% [10–12]. Recent studies have highlighted the same regarding UCBT [8]. Early attempts at introducing haploidentical family donor as an alternate donor had failed miserably. Not unexpectedly, severe alloreactivity or graft rejection dominated the outcome and the concept of allograft from a HFD was not thought to be feasible [13].

## 4. Megadoses of Purified CD34+ Cell Infusion: The Door Opened but Questions Remained

The breakthrough came from murine experiments demonstrating the ability of megadoses of CD34+ cells to engraft across major HLA barriers [14–16]. This was translated to clinical reality by the group from Perugia when they reported 95% engraftment with virtually no serious graft-versus-host disease (GVHD) without employment of GVHD prophylaxis, in patients with advanced leukemia [17]. This was possible due to advent of growth factor mobilized peripheral blood stem cell (PBSC) collection which enabled collection of large amounts of CD34 cells which was not hitherto possible from marrow grafts. The other advancement of technology provided the ability to purify CD34 cells via immunomagnetic techniques drastically reducing the T and B cell content of the graft. This approach was based on infusing CD34 cells in excess of 10 × 10^6^/kg with a CD3 cell inoculum of <1 × 10^5^/kg. In a pilot study on haploidentical HSCT with CD34 selected PBSC graft following myeloablative and immunoablative conditioning, Aversa et al. documented sustained engraftment in 41/43 patients with advanced leukemia without acute or chronic GVHD and 28% long term disease-free survival (DFS) [17]. Importantly, no pharmacological GVHD prophylaxis was employed. The study population included both adults and children with an age range of 4 to 53 years. However, the major drawback of this approach was delayed immune reconstitution resulting in mortality from opportunistic infections in about 40% of the patients. The reconstitution of CD4 T cells was delayed beyond 12 months in the surviving patients. In a study on 39 children employing a similar approach, 36 patients engrafted promptly with little or no GVHD [18]. The DFS was 28% and TRM was 34%. Interestingly, immune reconstitution (IR) was noted to be better in those receiving >20 × 10^9^/kg CD34 cells. Subsequent studies by the Perugia group showed further improvement in outcome over the next decade, but TRM remained a major concern which was attributable to delayed IR [19–21]. Two studies from the UK highlighted similar findings with better results in patients in CR than those who were not in remission [22, 23]. The outcome with this approach was remarkably better in patients with AML as compared to those with ALL [19].

An EBMT Pediatric Disease Working Party survey on 127 children with ALL transplanted between 1995 and 2004 revealed some interesting facts [24]. They found that transplants carried out by centres performing more than 231 allografts in the specified period with a median of 8 HFD yielded a DFS of 39% compared to only 15% in those performing less than 231 allografts with a median of one HFD transplant. There was a trend towards lower relapse incidence (RI) and DFS amongst those receiving a higher dose of CD34 cells. These findings highlighted the fact that T cell depleted (TCD) HFD transplantation was a technically demanding procedure requiring experience and the results heavily depended on the CD34 cell content of the graft. The other major hindrance for its universal application was the high TRM associated with delayed IR. Whilst the major centres performing such procedures develop protocols and expertise in managing these complications, the ones performing TCD HFD transplants only occasionally were unlikely to achieve similar results.

## 5. Natural Killer (NK) Cell Alloreactivity: A New Kid in the Block

The focus of GVHD and GVL had remained on T cells until Ruggeri et al. highlighted the impact of natural killer (NK) cells in reduction of relapse in AML following CD34 selected PBSC grafts from haploidentical donors [25]. Since then, several groups have reported on the impact of NK cells in shaping the outcome of both haploidentical family donor and unrelated donor transplantation. The opinion has often been divided on this issue [26–28]. The last decade has witnessed an enormous effort in the understanding of NK cell biology within the context of allogeneic hematopoietic cell transplantation (HCT).

NK cells kill their target through direct cytotoxicity by engaging one or more activating receptors. However, the activating receptors are believed to be under the negative feedback control from inhibitory killer immunoglobulin-like receptors (KIRs). Cytotoxicity of NK cells in the steady state is under the constant negative feedback from inhibitory KIRs through binding to Self-Class 1 MHC molecules. Several key KIR genes have been identified along with their putative ligands, whilst others remain unidentified. Biallelic polymorphism in HLA-C (positions 77 and 80 of heavy chain) denoted as C_1_ or C_2_ and restricted polymorphism in HLA-B (positions 77–83 in heavy chain) denoted as BW4 have been identified as ligands for KIR 2DL2/3, 2DL1, and 3DL1, respectively [29].

When NK cells from biallelic donor (C_1_ and C_2_), for example, fail to find one of the alleles (C_1_ or C_2_) in the recipient, a subset of donor NK cells tend to lose the inhibitory feedback and target the host hematopoietic cells vis-a-vis the leukemia cells in cytotoxic killing. This phenomenon (missing self-theory) was described by the Perugia group as the key event responsible for the cure of high risk leukemia following CD34 selected haploidentical graft [29, 30]. Several other models of NK alloreactivity have been postulated, yet none have been proven beyond surrogacy in the clinical setting [29–32].

In recent years, the focus has shifted to the repertoire of activating genes in the donor NK cells. Sivori et al. reported on the beneficial outcome of donor KIR2DS1 expression in conjunction with C2 allele in the recipient [33]. Furthermore Cooley et al. showed that KIR haplotypes and the specific genes related to B haplotype in the donor at centromeric or telomeric positions might have a favourable impact on the outcome of both unrelated and haploidentical HCT [34].

## 6. Manipulating the Graft Further: Positive versus Negative Selection

The seminal findings on NK alloreactivity along with development of immunomagnetic cell selection gave researchers in the field the options to rid the graft of CD3 and CD19 cells, leaving behind CD34, CD56, and other cell types [35]. The Tuebingen group reported on 46 children undergoing HFD HSCT with CD3+/CD19+ depleted graft in 2014 [36]. The engraftment was 88% with 20% TRM at 5 years. However, the incidences of both acute and chronic GVHD were higher with this approach, unlike that witnessed with CD34 selected grafts. The same group studied NK cell reconstitution in 59 patients undergoing CD3/19 depletion as compared to 42 patients undergoing CD34 selection [37]. They observed superior NK cell recovery and cytotoxicity with the former approach.

However, despite achieving a DFS of 45% to 80% when children were in CR, both TCD approaches were associated with dismal outcomes in more advanced diseases [18, 22, 36–38]. Employment of other TCD approaches in HFD transplantation for children with advanced leukemia did not result in improved outcome [39].

Further refinement of this approach took place with a new TCD method that removes *αβ*+ T lymphocytes via a biotinylated anti-TCR*αβ* antibody followed by an anti-biotin antibody conjugated to magnetic microbeads while retaining TCR *γδ*+ T lymphocytes, natural killer (NK) cells, and other cells in the graft [40]. This approach was based on the fact that the TCR*αβ* T cells were primarily responsible for GVHD and that TCR *γδ* T cells had potent antileukemia and antipathogen activity which, coupled with NK cells in the graft, would boost both antitumor and anti-infective potency of the graft. This approach has yielded excellent results in children with nonmalignant diseases and in those with acute leukemia in CR [41–44]. The IR was accelerated with this approach compared to the previous ones. The incidence of both acute and chronic GVHD remained low more akin to the CD3/CD19 depletion approach. However, the outcome of children not in CR remained dismal [43].

Another innovative approach from the Perugia group has taken graft manipulation a level further [45]. In accordance with the animal studies, they infused CD4+ CD25+ FoxP3+ regulatory T cell subpopulation (Tregs) on day −4 at 2 × 10^6^/kg following myeloablative conditioning [46]. This was followed by infusion of >10 × 10^6^/kg CD34 cells on day 0 along with 1 × 10^6^/kg conventional T cells. This study was exclusively in adults and resulted in a DFS of 53% in patients with high risk leukemia, primarily in remission [46]. The authors claimed that this approach might reduce GVHD and yet augment the GVL effect. This approach is exciting but expensive and labor intensive.

Despite the encouraging results of TCD based approaches, two major caveats remain. Firstly, the approaches are technically demanding and expensive limiting its global application. Second, TCD based HSCT has uniformly yielded abysmal results in more advanced leukemia, particularly if not in remission [47].

## 7. Unmanipulated Haploidentical HSCT: Changing the Paradigm in Adults, but What about Children?

Two major approaches to HFD HSCT without graft manipulation in adults have changed the approach and outlook towards haploidentical transplantation in the last 5 years. The first approach pioneered by the Peking University group employed myeloablative conditioning with combined G-CSF stimulated marrow and PBSC grafts along with multiagent GVHD prophylaxis [48, 49]. Outcome data on 1210 transplants were reported in both adults and children with mostly ALL and AML with an impressive DFS of 67% and a NRM of 17% [50]. The incidences of acute and chronic GVHD were 40% and 50%, respectively. The RI was only 17%. The same group reported on the outcome of 212 children with a median age of 15 years with both AML and ALL [51]. They reported 100% engraftment with a NRM of 15% in those transplanted in CR1/CR2, but 25–40% in those beyond CR2. The incidences of both acute and chronic GVHD were similar to those reported in the combined population, but grades 3-4 GVHD which occurred in 15% of patients was identified as a risk factor for NRM. The RI was 7.2% and 19% in CR1 for AML and ALL, respectively, but was 2–4-fold higher beyond CR1. The overall DFS was 73% for AML and 57% for ALL. In those beyond CR2, the DFS was 42% for AML and 22% for ALL. These results compare favourably with TCD approaches reported thus far. Not surprisingly, the incidences of both acute and chronic GVHD were much higher with this approach.

The other approach which was pioneered by the Johns Hopkins group involved use of posttransplantation cyclophosphamide (PTCY) [52]. This simple but unique concept is based on the fact that activated T cells are susceptible to high dose cyclophosphamide if administered in the window of 72 hours after graft infusion. The hematopoietic stem cells as well as quiescent T cells are spared of the cytotoxic effects of PTCY due to higher amount of aldehyde dehydrogenase [53, 54]. It was shown in preclinical as well as the subsequent clinical studies that this approach resulted in 90% engraftment with very low incidences of both acute and chronic GVHD [55]. These studies were carried out in adults and the conditioning was nonmyeloablative (NMA) with marrow as the source of graft. The GVHD prophylaxis consisted of mycophenolate mofetil (MMF) for 35 days and tacrolimus for 180 days. In those grafted in CR1, the results were encouraging, but the ones with more advanced disease experienced very high incidences of relapse [56]. Subsequent studies on PTCY based HFD HSCT employing myeloablative conditioning reported better DFS with no significant increase in GVHD or NRM [57, 58]. At the same time, several groups have used PBSC graft instead of BM and the outcomes have been similar in terms of engraftment and NRM with some increase in acute GVHD [59–61]. Thus, these studies have established PTCY based haploidentical HSCT as a frontrunner when it comes to alternate donor HSCT, to the extent that many argue in favour of PTCY based HFD HSCT ahead of MUD or UCBT [62–64].

Despite the impressive results in adults, the literature has been largely silent on the use of PTCY in children. One study from Japan employed a modified PTCY based approach on day +3 alone and GVHD prophylaxis with steroids and tacrolimus in 15 children, 9 of whom had advanced leukemia [65]. They reported a higher incidence of graft failure with lower conditioning intensity. Although 46% of the patients achieved a CR, the long term outcome remained dismal.

We had carried out a pilot study with PTCY based haploidentical PBSC transplantation on 20 children with advanced leukemia, 13 with refractory or relapsed AML and 7 with high risk ALL in CR1 [66]. A myeloablative conditioning with Fludarabine, Busulfan, and Melphalan was employed and GVHD prophylaxis consisted of MMF for 14–21 days and cyclosporine for 60 days with further 2 weeks of tapering. All engrafted promptly with 35% experiencing grade 2–4 GVHD and 5% having mild chronic GVHD. NRM was 20% at 1 year and this was associated with grade 3-4 GVHD, similar to that reported by the Chinese group [51]. However, it was of note that grade 3-4 GVHD occurred exclusively in those below the age of 10 years in our study. The above-mentioned study from Japan also documented GVHD in 6/8 evaluable patients below the age of 10 years [65]. In addition, we also experienced a higher incidence of early alloreactivity in the form of hemophagocytic syndrome (HPS) in children below 10 years of age [67].

## 8. Why Are Younger Children at a Higher Risk of Early Alloreactivity following PTCY Based Haploidentical HSCT?

This finding is indeed intriguing and counterintuitive on the face of it. The high incidence of early alloreactivity in the younger children undermines the basic principle of the PTCY approach and contradicts the prevailing concept that GVHD occurs with increasing age rather than the other way around. The relative contents of CD34+ cells and CD3+ T cells in our study were similar in both younger and older children and hence the higher T lymphocyte content of PBSC graft is unlikely to be solely responsible for the disparate outcome in the younger children [66]. Based on these findings, we hypothesised that the possible reason for early alloreactivity could be related to the failure of elimination of the alloreactive T cells by PTCY in younger children. To support this hypothesis, the pharmacokinetic (PK) studies on CY metabolism in children have been shown to be extremely variable [68]. In a study on 38 children between the ages of 2 and 15 years, there was significant interpatient variability as well as variable activation of CY to its active metabolites [69, 70]. A pharmacokinetic study of high dose CY in children above the age of 10 years undergoing myeloablative conditioning for solid tumours did not reveal any impact of age on clearance or the volume of distribution of CY [71]. Extrapolating from the pharmacokinetic studies, this phenomenon might be explained by the reduced efficacy of PTCY in clearing alloreactive T cells in younger children, due to the variable metabolism of the drug in younger age group. Whether the alloreactivity would be less with marrow grafts is unclear due to the lack of data on the same. These findings once again serve as a reminder not to consider children as mere smaller adults and a regimen deemed successful in adults might not necessarily yield similar results in children.

## 9. Choice of Graft for Children in CR1 Lacking a Matched Donor?

In those in whom an allograft is recommended in CR1, traditionally, a MUD or UCBT from a cord unit with high cell dose and ≤2 allele mismatches would generally be preferred for both AML and ALL. In conventional algorithm, a TCD graft from a HFD would be considered appropriate if none of the above is available. The relevant studies on HFD HSCT in children have been summarised in Tables 1 and 2. Whilst this approach is feasible and can produce impressive results in experienced hands, the procedure remains challenging to most of the world due to financial and technical demands associated with it. However, the cost of procuring a cord or a MUD graft is even more and the absence of GVHD and its prophylaxis or treatment following TCD HFD graft largely balances out the upfront financial burden in the long term along with an improved quality of life due to lack of immunosuppression and chronic GVHD. The best results with this approach are obtained in patients in complete remission, CR1 or CR2, rather than those not in CR or beyond CR2 [18, 20, 36, 39, 43]. The newer approaches to TCD such as TCR*αβ* depletion might be more appropriate than CD34 selection due to the poor immune reconstitution associated with the latter resulting in significant infection associated mortality [20, 39, 43, 72]. However, the data on the former is scanty and follow-ups are short to allow any definitive verdict in favour of either. Furthermore, NK cell alloreactivity plays an important role in reducing relapses for myeloid malignancies following TCD grafts in the HFD setting [32, 73]. The same is not established unequivocally in the context of ALL [73]. Some studies have suggested that NK cell alloreactivity might be effective in T cell ALL as well, whilst another study suggested an improved outcome in childhood ALL with a donor NK cell KIR B haplotype with higher B score [74].

The preferred modality of graft manipulation would be subject to the experience of individual centres with the main thrust on administering high number of CD34 cells, preferably in the range of 15–20 × 10^6^/kg. An NK alloreactive donor would be preferred as would be a maternal donor, if the graft is T cell depleted [75]. The issue of donor NK haplotype and B score might be relevant but remains uncertain pending further studies. However, if more than one NK alloreactive HFD is available, choosing one with a B haplotype and/or higher B score might be preferred. Although the data on NK cell alloreactivity is more robust in HFD transplants for AML, the limited data should not preclude the choice of the same in ALL.

If TCD is not feasible due to technical or financial reasons, should one opt for an unmanipulated graft and if so, what should dictate the choice of the donor? Given the limited data on non-TCD approaches, the recommendations would be more tailored to the individual situation. The study by the Chinese group has yielded impressive results in both AML and ALL in CR1. However, data is not available from other centres employing a similar approach and it remains unclear if the results would be similar in other ethnic groups. This is exemplified by a much higher incidence of HPS following both UCBT and HFD HSCT from Asia as compared to Europe and Northern America [67, 76]. The data on PTCY based approach is limited, but early data indicates that this approach is best limited to children above the age of 10 years due to a higher risk of early alloreactivity [66].

The next issue that needs to be addressed is related to the choice of the haploidentical family donor. If the former approach is chosen, the choice of donor might be more definitive as donor issues have been extensively studied by the researchers from Peking University [50]. Interestingly, in direct contradiction to the data from TCD HFD [75], maternal donors were found to be associated with poorer outcomes. Three factors stood out in this analysis, donor gender, donor age, and noninherited maternal antigen (NIMA) mismatch. Thus, a NIMA mismatched younger male sibling would be a preferred donor followed by the father over mother or a sister. The same group had shown a detrimental effect of NK cell alloreactivity on the outcome, which was again contrary to the findings from TCD approach [77]. The same, however, cannot be extrapolated to other forms of unmanipulated HFD grafts and, pending further studies, a NIMA mismatched sibling donor might be a reasonable option. However, given the increased number of single child nuclear families, one might be left to choose between the parents. The Johns Hopkins group had shown that there might not be an impact of NK cell alloreactivity in the context of NMA PTCY based haploidentical transplantation [78]. Rather, a HFD with NK B haplotype might yield better results. This again remains unproven in the pediatric setting following myeloablative conditioning.

The current excellent results of TCD haploidentical HSCT could challenge the current hierarchical algorithm of alternate donor choice of MUD and UCBT in preference to HFD grafts, especially when the financial implications of the latter are more favourably balanced in the long term. It would not be unwise to assume that continued advances in the field of HFD HSCT might make this form of alternate donor HSCT the preferred option in the near future.

## 10. The Choice of Graft beyond CR1/CR2

The results of TCD approaches have been uniformly dismal even with newer methods of graft manipulation in these patients [43, 47] and a non-TCD approach might be preferred. Given the high risk of treatment failure, higher incidence of both acute and chronic GVHD following an unmanipulated graft might be more acceptable. Similar to TCD approaches, the choice would be centre specific with the main aim of the regimen directed at reducing both relapse and NRM. However, if a TCD approach is employed, this needs to be combined with immunotherapy. Whether infusion of NK cells or even *γδ*T cells can improve the outcome remains to be seen but poses an exciting area of research [41, 79, 80]. The other approach being studied is the use of suicide gene modified T cells [81, 82]. Thus, it might be prudent to enroll such patients in one of the trials employing any of these approaches. At our centre, we continue with Flu-Bu-Mel conditioning and PBSC graft with PTCY and attenuated courses of both MMF and CSA in those above 10 years old. In those under the age of 10 years, we are currently enrolling patients with relapsed refractory leukemia in a study exploring inhibitors of T cell activation with PTCY to prevent early alloreactivity or TCD grafts with active immunotherapy.

## 11. Optimizing NK Cell Mediated GVL Effect in Unmanipulated Haploidentical HSCT

NK cell alloreactivity is unequivocally demonstrable following TCD haploidentical HSCT and yet has not been discernable with an unmanipulated graft. This paradox has never been addressed but undoubtedly deserves a closer look. There could be several explanations for this phenomenon. Ligand mismatches are not the only prerequisite for realising the antileukemia effect of alloreactive NK cells. Studies on HFD transplantation with unmanipulated graft have employed MMF as GVHD prophylaxis and have routinely employed G-CSF after transplant. Both of these interventions compromise NK cell cytotoxicity [83, 84] and so does sirolimus [85]. On the other hand, CSA probably does not impair NK cell activity and might even augment it [85, 86].

High dose CY has also been shown to enhance NK cell activation. Administration of high doses of CY improved the antitumor effect of IL-2 activated NK cells in animal models [87]. The cytotoxic activity of NK cells was increased by over 300% when they were incubated with CY. This effect was demonstrable after 2 hours and maximised after 8 hours of incubation [88]. In a clinical study on adoptive immunotherapy with haploidentical NK cells in patients with refractory AML, the use of high dose CY was associated with increased expansion of donor NK cells [89]. This was attributed to a marked rise in endogenous IL-15, a phenomenon not witnessed with low dose CY.

Thus, the combination of PTCY and CSA might provide the ideal platform to exploit the antileukemic potential of alloreactive NK cells, if the use of MMF or G-CSF could be limited. Furthermore, the use of PBSC graft rather than marrow might contribute to this phenomenon.

## 12. Conclusion

Haploidentical HSCT has come a long way since the initial failures in the 1980s [90]. The concepts of both the veto effect of CD34 cells when infused alone in large amounts and the utilisation of metabolic principles of cyclophosphamide in eradicating activated T cells immediately after transplantation have ushered a new era in alternate donor transplantation. Newer methods of graft manipulation with adoptive immunotherapy might pave the way for greater successes in the field of HFD transplantation for children with acute leukemia. At the same time, improving on the approaches to unmanipulated haploidentical HSCT is essential to realise the global potential of this procedure.

## Figures and Tables

**Table 1:** Outcome of T cell depleted haploidentical transplantation for children with acute leukemia.

Ref.	Patients with AL (total)	Age range (years)	Disease status	Conditioning	Graft manipulation	Graft composition		Engraftment (%)	Acute GVHD (%)	Chronic GVHD (%)	NRM (%)	Relapse (%)	Overall survival (%)
						CD34 (×10^6^)	CD3 (×10^7^)						
Aversa et al. (1998) [17]	43	4–53	Advanced	FLU/TT/ATG/TBI	CD34 selection	14	2.7	95.3%	None	None	40%	AML: 13% ALL: 63%	AML: 36% ALL: 17%

Handgretinger et al. (2001) [18]	21 (39)	(0.5–18)	NR = 9 CR = 12	MA	CD34 selection	20.7	1.5	92.3%	5%	None	28%	33%	NR: 14% CR: 39%

Goldman et al. (2000) [39]	52	1–19	AML Rel./Ref.	TBI: 40 Non-TBI: 12	Bone marrow CD2 depletion	MNC×10^8^	3.04	71%	44%	None	71%	26%	2%

Ortín et al. (2002) [23]	16 (21)	2–16	CR	CY/TBI	CD34 selection	8.5	5.6	100%	43%	25%	4.8%	18.7%	81.3%

Marks et al. (2006) [22]	34	1–16	CR 18 NR 16	CY/TBI/Campath/ATG	CD34 selection	13.8	0.3–5.2	91.7%	29%	12%	29.4%	CR: 13% NR: 100%	26% AML CR: 28% ALL CR: 38%

Klingebiel et al. (2010) [24]	127	0.6–16	ALL NR 25 CR 102	MA	CD34 selection	12.3	5.0	91%	37%	16.7%	37%	36%	CR: 22–39% NR: 0%

Leung et al. (2011) [38]	38	<16	NR 5 CR 30	TBI: 20Non-TBI: 15	CD34 selection: 13 CD3 depletion: 17 Others: 5	NA	9–44	NA	25.7%	NA	23.9%	14.7%	<2002: 19% >2002: 88%

Lang et al. (2014) [36]	46	1.1–23.7	NR 20 CR 26	FLU/TT/MEL +ATG/OKT3	CD3/19 depletion	14.5	0.59	81.6%	26%	21%	10.8%	38%	CR: 31% NR: 20%

Lang et al. (2015) [43]	29 (41)	<16	NR 9 CR 20	NA	TCR*α*/*β* and CD3 depletion	14.9	1.69	88%	24%	18%	NA%	47.2%	CR1–CR3: 100% NR: 0%

AL: acute leukemia; ALL: acute lymphoblastic leukemia; AML: acute myeloid leukemia; ATG: antithymocyte globulin; CR: complete remission; CY: cyclophosphamide; GVHD: graft-versus-host disease; FLU: Fludarabine; MA: myeloablative conditioning; Mel.: Melphalan; NR: not in remission; NRM: nonrelapse mortality; Ref.: refractory; Rel.: relapsed; TBI: total body irradiation; TT: thiotepa.

**Table 2:** Outcome of haploidentical transplantation for children with acute leukemia without T cell depletion.

Ref.	Number of patients with AL (total)	Age range (years)	Disease status	Conditioning	Graft composition		GVHD prophylaxis	Engraftment (%)	Acute GVHD (%)	Chronic GVHD (%)	NRM (%)	Relapse (%)	Overall survival (%)
					CD34 (×10^6^)	CD3 (×10^7^)							
Liu et al. (2013) [51]	212	3–18	NR = 24 CR = 188	AraC/BU/CY Semustin/ATG	2.5	1.88	Multiagent	100%	48.8%	40.1%	<2008: 16.8% >2008: 12.2%	<2008: 28.3% >2008: 17.5%	<2008: 61.1% >2008: 71.5%

Sawada et al. (2014) [65]	9 (15)	2–17	Ref./Rel.: 7 CR = 2	FLU/MEL	NA	NA	PTCY based	80%	55.6%	NA	28.5%	57.1%	Ref./Rel.: 14.2% CR = 100%

Jaiswal et al. (2016) [66, 67]	20	2–20	AML Rel./Ref.: 13 ALL CR: 7	FLU/BU/MEL	7.5	6.85	PTCY based	100%	35%	5%	20%	25.7%	64.3%

AL: acute leukemia; ALL: acute lymphoblastic leukemia; AML: acute myeloid leukemia; ATG: antithymocyte globulin; BU: Busulfan; CR: complete remission; CY: cyclophosphamide; GVHD: graft-versus-host disease; FLU: Fludarabine; MA: myeloablative conditioning; Mel.: Melphalan; NR: not in remission; NRM: nonrelapse mortality; Ref.: refractory; Rel.: relapsed; TBI: total body irradiation; TT: thiotepa.
